# A Bayesian network meta-analysis of non-pharmacological interventions for neonatal pain management: a clinical effectiveness comparison

**DOI:** 10.3389/fped.2025.1547308

**Published:** 2025-05-22

**Authors:** Lingxue Xu, Lali Xiang, Lihui Pan, Peipei Xue, Juan Li, Yurong He, Hongyan Liu, Yuwei Hu, Bo Zheng

**Affiliations:** ^1^ NICU, Yuhuan People's Hospital, Taizhou, China; ^2^ Global Visiting Scholar Program, UNC of Greensboro, Greensboro, NC, United States

**Keywords:** non-pharmacological, network meta-analyses, pain mamgement, neonatal infant pain scale, randomized clincial trial, SUCRA, newborn

## Abstract

**Background:**

Newborns are particularly vulnerable to pain, and non-pharmacological methods are frequently employed for pain management due to their lack of side effects. However, there is a lack of comprehensive comparison and ranking of the effectiveness of various non-pharmacological interventions.

**Objective:**

To evaluate the effectiveness of non-pharmacological interventions and to determine whether differences exist in the efficacy of various interventions.

**Design:**

Systematic review and network meta-analysis.

**Data source:**

RCTs studies in MEDLINE, EMBASE, Web of Science, Cochrane Central Register of Controlled Trials from inception to November 1, 2024.

**Review methods:**

Up to November 1, 2024, we conducted a comprehensive search across four databases to identify studies meeting our inclusion criteria. A Bayesian model was employed for the analysis, and heterogeneity was quantified using random-effects standard deviation (RESD), *τ*², and I² statistics. The certainty of the synthesized evidence was evaluated using the Grading of Recommendations, Assessment, Development, and Evaluation (GRADE) approach. This study protocol has been registered with PROSPERO.

**Results:**

Initially, we identified 20 citations and included 59 trials involving 2,028 participants for network meta-analysis. Due to many interventions being supported by only one or two original studies, we excluded interventions with fewer than three studies. Ultimately, we identified 13 citations, including 31 trials with a total of 1,141 participants. Regarding efficacy, several interventions were found to be effective. Breast milk (BM), sweet taste (ST), Yakson touch (YT), swaddling, and heel warming (HW) demonstrated significant effectiveness, with mean differences (MDs) in NIPS scores as follows: BM vs. control, −1.71 [95% credible interval (CrI): −2.29, −1.17]; ST vs. control, −1.35 (CrI: −2.13, −0.52); YT vs. control, −1.41 (CrI: −2.09, −0.74); swaddling vs. control, −0.65 (CrI: −1.23, −0.13); and HW vs. control, −0.53 (CrI: −0.89, −0.01). In pairwise comparisons between interventions, significant efficacy differences were observed: BM vs. HW, −1.89 (CrI: −2.70, −1.05); BM vs. non-nutritive sucking (NNS), −1.89 (CrI: −2.70, −1.05); BM vs. ST, −0.88 (CrI: −1.61, −0.11); BM vs. YT, −0.82 (CrI: −1.56, −0.03); BM vs. swaddling, −1.59 (CrI: −2.20, −0.78); NNS vs. ST, 0.98 (CrI: 0.11, 1.89); NNS vs. YT, 1.06 (CrI: 0.12, 2.03); and HW vs. YT, 0.89 (CrI: 0.23, 1.69). Notably, NNS was not found to be effective. The Surface Under the Cumulative Ranking Curve (SUCRA) analysis suggested that BM may be the most effective non-pharmacological intervention for neonatal pain management. SUCRA rankings for the interventions were as follows: BM > ST > YT > swaddling > HW > NNS > control. However, the certainty of the evidence ranged from moderate to very low. Heterogeneity assessments indicated a random-effects standard deviation (RESD) of 0.28 (CrI: 0.04, 0.73) in the consistent model and 0.36 (CrI: 0.01, 1.36) in the inconsistent model, with *I*² = 100% and *τ*² = 2.22.

**Conclusion:**

Given the limitation of high heterogeneity, this study should be regarded as a clinical effectiveness comparison. Among the included interventions, breast milk (BM), sweet taste (ST), Yakson touch (YT), heel warming (HW), and swaddling were found to be efficacious, while non-nutritive sucking (NNS) was not effective. The top three interventions, based on ranking, were BM, YT, and ST. However, some effects should be interpreted with caution, as they are derived from small sample sizes. Future research should focus on identifying factors associated with individual responses through large, multicenter studies.

**Implications for Nursing Management:**

Findings will inform nurse managers of an ideal environment for the non-pharmacological pain management for newborn.

**Systematic Review Registration:**

https://www.crd.york.ac.uk/PROSPERO/view/CRD42023399924, PROSPERO CRD42024567338.

## 1 Introduction

Pain is a unique sensation with both emotional and psychological dimensions. The development of the nervous system occurs in distinct phases: the nociceptive system is fully developed by 20 weeks of gestation, while cortical pain perception emerges when the thalamic tract completes its connection to the dorsal horn, around 24 weeks of gestation. Several studies have demonstrated that from 25 weeks of gestation, infants exhibit a clear response in the contralateral cortex following a noxious stimulus, as evidenced by electroencephalogram activity and cerebral oxygenation measurements during the stimulus. These early experiences of pain may contribute to the development of psychological and psychiatric disorders, such as depression, anxiety, and an increased vulnerability to chronic pain (1, 2). Avoiding painful procedures during the neonatal and infant stages contributes to harmonious neurodevelopment and helps prevent long-term adverse consequences. Pain management methods can be categorized into two main types: pharmacological and non-pharmacological (3). Pharmacological methods for managing pain during short-term painful interventions have not been approved due to concerns about long-term effects and potential side effects, including itching, respiratory depression, nausea, and vomiting. In contrast, non-pharmacological pain management encompasses techniques or interventions that do not involve the use of drugs or medications. These approaches focus on physical, psychological, or behavioral strategies to reduce pain perception and enhance overall quality of life, without the risk of side effects. Consequently, non-pharmacological methods are more suitable for pain relief in infants, given their short-term nature and better tolerance among this population (4). In this context, non-pharmacological methods such as sweet taste (ST, including sucrose and glucose solutions), breast milk (BM), white noise (WN), non-nutritive sucking (NNS), swaddling, and Yakson touch (YT) can be effectively utilized to prevent pain and reduce neonatal distress during painful procedures (5).

The Neonatal Infant Pain Scale (NIPS) was developed in the late 1980s by Dr. Diane L. Lawrence to provide a reliable, valid, and straightforward tool for assessing pain in neonates. Designed as a behavioral-based scale, NIPS enables clinicians to quickly evaluate the presence and severity of pain in neonates, addressing the critical need for a pain assessment tool in this population, particularly in situations where infants cannot verbally express their discomfort. In this study, we utilized the Neonatal Infant Pain Scale (NIPS) as the basis for analyzing pain-related studies.

We selected the heel stick procedure for this research due to its standardized nature, in contrast to other procedures such as venipuncture, intravenous infusion, or endotracheal intubation, which exhibit considerable variation in duration. Focusing on a standardized procedure helped minimize variability and ensure consistent comparisons across studies.

The aim of this network meta-analysis was to evaluate the existing literature on non-pharmacological approaches for managing pain during the heel stick procedure, as assessed using the Neonatal Infant Pain Scale (NIPS). This analysis seeks to provide valuable insights into current evidence and offer recommendations for future clinical practice.

## 2 Methods

### 2.1 Inclusion and exclusion criteria

Studies were included in our network meta-analysis if they met the following criteria: (1) compared non-pharmacological interventions for pain prevention in neonates, (2) assessed pain using the Neonatal Infant Pain Scale (NIPS), (3) involved healthy neonates or those with APGAR scores >7 (no asphyxia), (4) focused on the heel stick procedure, and (5) were randomized controlled trials (RCTs).

Studies were excluded if they (1) lacked data suitable for meta-analysis, such as providing only the range of minimum and maximum values or data that could not be extracted, or (2) involved infants under the influence of drugs like analgesics, sedatives, or corticosteroids with residual effects during the intervention. There were no language restrictions for study inclusion.

### 2.2 Search strategy and selection criteria

We conducted a systematic review and network meta-analysis. Searches were performed in the Cochrane Central Register of Controlled Trials, EMBASE, MEDLINE, and Web of Science from their inception through November 1, 2024.

The search strategy employed the following terms: *((neonatal infant pain scale [Title/Abstract]) AND ((neonate [Title/Abstract]) OR (newborn [Title/Abstract])) AND (“heel”[Title/Abstract]))*. We did not examine reference lists from clinical trial reports, meta-analyses, or systematic reviews to identify additional relevant studies.

Eligible studies included randomized controlled trials (RCTs) comparing one non-pharmacological intervention with another or with a control group as monotherapy for pain management in neonates (≤28 days old, both sexes). However, some included studies were not blinded to the assessors, which may have introduced bias and potential distortion in the scoring system.

### 2.3 Study selection and data collection

Two investigators (Lihui Pan and Lali Xiang) independently screened the titles, abstracts, and full-text articles for inclusion, utilizing standardized data extraction forms. Any disagreements were resolved through discussion. For sweet taste interventions, studies involving sucrose and glucose methods were combined, irrespective of dose or concentration parameters. In cases where a study reported separate data points, such as baseline NIPS, NIPS during the procedure, or NIPS after the procedure, we calculated and analyzed the mean difference and standard deviation (SD) of NIPS scores (refer to Supplementary File S1 for the formula).The following information was extracted from each trial: (1) first author, (2) year of publication, (3) number of arms (included in the network meta-analysis and study), (4) country, (5) interventions, (6) sample size, (7) baseline characteristics (postnatal age, gestation week, weight, sex, Apgar score), (8) NIPS. And (9) risk of bias. In this study, the control group was defined as the group that did not receive any intervention. Corrections to some papers are detailed in Table 1. For missing data, we contacted the authors of trial reports or extracted data from Figures using the WebPlotDigitizer tool (online).

**Table 1:** The basic information of primary studies.

Study ID	Year	No. of arms included in NMA	No of total study arms	Country	Intervention	No. randomised	Postnatal age, (d) (mean ± SD)	Gestation week (mean ± SD)	Birth weight (g)	Nips score (mean ± sd)	Apgar 1 min	Apgar 5 min	Male (%)	Risk of bias
										Before = before procedure				
										During = during procedure				
										After = after procedure				
Lan (6)	2021	3	3	China	Touch + vc	40	2–3	39.42 ± 1.00	3,121 ± 347	Total: 0.61 ± 0.95	7.88 ± 0.33	9.0 ± 0.00	42.5	High
					Touch + vc + breastmilk-odor	40	2–3	39.07 ± 1.09	3,109 ± 375	Total: 0.46 ± 0.79	7.88 ± 0.33	8.9 ± 0.15	55.0	
					Touch + vc + breastmilk-odor + EBM	40	2–3	38.91 ± 0.97	3,070 ± 340	Total: 0.42 ± 0.82	7.88 ± 0.33	8.9 ± 0.15	50.0	
Tasci (7)	2020	2	2	Türkiye	FM-odor + massage	42	<1	38–42	2,500–4,000	Before: 0.12 ± 0.40	>8	>8	Unclear	High
										During: 4.62 ± 1.87				
										After: 1.83 ± 0.96				
					BM-odor + massage	42	<1	38–42	2,500–4,000	Before: 0.02 ± 0.15	>8	>8	Unclear	
										During: 2.00 ± 1.33				
										After: 0.36 ± 0.66				
Akcan (8)	2016	2	2	Türkiye	Lavender-odor	27	Unclear	Unclear	3,402.2 ± 384.1	Before: 0.04 ± 0.13	Unclear	9.96 ± 0.19	51.9	Unclear
										During: 2.91 ± 2.47				
										After: 0.87 ± 1.16				
					Breastfed- odor	24	Unclear	Unclear	3,336.2 ± 351.5	Before: 0.0 ± 0.0	Unclear	10.00 ± 0.00	50	
										During: 3.31 ± 2.39				
										After: 0.67 ± 0.90				
					Amniotic Fluid-odor	26	Unclear	Unclear	3,401 ± 223.7	Before: 0.0 ± 0.0	Unclear	10.00 ± 0.00	46.2	
										During: 3.90 ± 2.47				
										After: 0.71 ± 1.02				
					Water-odor	25	Unclear	Unclear	3,401 ± 310	Before: 0 ± 0	-	10 ± 0	40	
										During: 5.20 ± 2.10				
										After: 1.02 ± 1.12				
Soltani (9)	2018	3	4	Iranian	BM	42	3–5	37–42	2,880 ± 950	Before: 0.19 ± 0.02	>9	>9	43	High
										Total: 5.52 ± 2.22				
					SSC	38	3–5	37–42	3,260 ± 420	Before: 0.20 ± 0.06	>9	>9	68.4	
										Total: 6.84 ± 1.96				
					ST	40	3–5	37–42	3,110 ± 460	Before: 0.18 ± 0.08	>9	>9	62.5	
										Total: 6.45 ± 1.88				
Wu (10)	2021	2	2	China	BM	48	12.20 ± 3.15	Unclear	3,290 ± 11	Total: 2.13 ± 0.25	Unclear	>8	62.5	High
					Control	48	12.15 ± 3.21	Unclear	3,215 ± 11	Total: 4.52 ± 0.58	Unclear	>8	58.3	
Ahn (11)	2006	3	3	Korea	ST	20	3.80 ± 1.00	39.14 ± 0.91	3,420 ± 430	During: 5.80 ± 2.01	Unclear	9.30 ± 0.70	50	Low
										Immediately after: 4.60 ± 2.11				
										3 min after: 1.70 ± 2.51				
					NNS	20	4.40 ± 0.90	39.26 ± 1.17	3,380 ± 410	During: 6.95 ± 0.22	Unclear	9.40 ± 1.00	45	
										Immediately after: 6.05 ± 1.46				
										3 min after: 3.90 ± 2.84				
					Control	20	3.80 ± 1.00	39.14 ± 0.93	3,310 ± 410	During: 6.65 ± 0.98	Unclear	9.30 ± 0.80	70	
										Immediately after: 6.00 ± 1.80				
										3 min after: 2.30 ± 2.63				
Yarahmadi (12)	2024	2	2	Iran	WN	40	<7	34.62 ± 1.39	1,999.25 ± 434.10	Before: 0.00 ± 0.00	8.22 ± 1.02	9.10 ± 0.84	55	Low
										During: 3.55 ± 0.84				
										After: 1.15 ± 0.84				
					SSC	40	<7	34.57 ± 1.21	1,972.25 ± 431.70	Before: 0.00 ± 0.00	8.22 ± 0.97	9.12 ± 0.82	57.5	
										During: 5.57 ± 0.95				
										After: 3.00 ± 0.98				
Apaydin Cirik (13)	2023	6	6	Türkiye	MV	31	1.00 ± 0.00	38.52 ± 0.85	3,108.87 ± 283.09	Before: 0.52 ± 1.36	Unclear	Unclear	45.2	H
										During: 5.23 ± 0.72				
										One minute after: 3.55 ± 0.62				
					WN	31	0.97 ± 0.18	38.77 ± 1.20	3,225.97 ± 472.11	Before:0.00 ± 0.00	Unclear	Unclear	61.3	
										During: 3.90 ± 0.75				
										One minute after: 1.74 ± 0.77				
					hold	30	1.07 ± 0.25	38.67 ± 0.76	3,350.40 ± 395.68	Before: 0.33 ± 0.92	Unclear	Unclear	70	
										During: 5.50 ± 0.57				
										One minute after: 3.73 ± 0.74				
					WN + hold	29	1.00 ± 0.00	38.38 ± 0.98	3,093.28 ± 300.35	Before: 0.03 ± 0.19	Unclear	Unclear	41.4	
										During: 3.34 ± 0.72				
										One minute after: 0.72 ± 0.59				
					MV + hold	28	1.00 ± 0.00	38.68 ± 0.94	3,275.00 ± 387.93	Before: 0.50 ± 1.40	Unclear	Unclear	50	
										During: 4.14 ± 0.89				
										One minute after: 3.14 ± 0.65				
					Touch^a^	29	1.03 ± 0.19	38.76 ± 0.87	3,405.86 ± 299.90	Before: 0.31 ± 0.66	Unclear	Unclear	82.8	
										During: 6.48 ± 0.51				
										One minute after: 4.97 ± 0.94				
Avan Antepli (14)	2022	2	2	Türkiye	Vibration	28	8.17 ± 1.72	38.71 ± 0.65	3,252.50 ± 332.21	Evaluated by the nurse	Unclear	Unclear	46.4	High
										before: 0.78 ± 1.89				
										15–20 s after: 2.89 ± 2.43				
										5 min after: 2.60 ± 2.80				
										Evaluated by the first specialist				
										Before: 1.00 ± 1.82				
										15–20 s after: 2.53 ± 2.08				
										5 min after: 2.60 ± 2.65				
										Evaluated by the second specialist				
										Before: 1.03 ± 1.97				
										15–20 s after: 2.96 ± 2.28				
										5 min after: 2.96 ± 2.91				
					Control	28	9.03 ± 2.42	38.82 ± 0.77	3,249.28 ± 332.07	Evaluated by the nurse	Unclear	Unclear	53.6	
										Before: 0.50 ± 1.23				
										15–20 s after: 5.71 ± 1.32				
										5 min after: 4.42 ± 2.71				
										Evaluated by the first specialist				
										Before: 0.46 ± 1.03				
										15–20 s after: 5.71 ± 1.53				
										5 min after: 4.46 ± 2.64				
										Evaluated by the second specialist				
										Before: 0.53 ± 1.13				
										15–20 s after: 5.67 ± 1.44				
										5 min after: 4.42 ± 2.68				
Inal (15)	2022	3	3	Türkiye	Swaddling	35	2–4	38.82 ± 0.98	3,378.28 ± 394.58	During:5.82 ± 0.92	Unclear	Unclear	51.4	High
										2 min after: 1.20 ± 1.45				
					Holding	35	2–4	39.22 ± 0.97	3,493.28 ± 487.69	During: 5.57 ± 1.24	Unclear	Unclear	48.6	
										2 min after: 0.60 ± 1.00				
					Control	35	2–4	39.02 ± 0.98	3,286.71 ± 493.61	During: 6.40 ± 0.91	Unclear	Unclear	48.6	
										2 min after: 1.94 ± 1.69				
Koç Özkan (16)	2019	2	3	Türkiye	Acupressure	46	1–2	Unclear	Unclear	During: 4.30 ± 2.25	Unclear	>7	37	Unclear
										1 min after: 1.46 ± 1.46				
					Massage	47	1–2	Unclear	Unclear	During: 3.95 ± 2.63	Unclear	>7	27.4	
										1 min after: 1.66 ± 1.66				
					Control	46	1–2	Unclear	Unclear	During: 6.04 ± 1.26	Unclear	>7	35.6	
										1 min after: 3.85 ± 1.37				
Aydin (17)	2019	3	3	Türkiye	Control	50	2–4	38.98 ± 0.96	3,241.10 ± 483.73	During: 6.42 ± 0.91	Unclear	Unclear	50	Unclear
					HW	50	2–4	39.14 ± 1.11	3,344.00 ± 448.67	During: 6.10 ± 1.07	Unclear	Unclear	50	
					BM	50	2–4	39.36 ± 1.01	3,394.40 ± 503.08	During: 4.44 ± 1.21	Unclear	Unclear	50	
Yilmaz (18)	2020	4	4	Türkiye	Control	40	2–4	38.92 ± 1.20	3,248.12 ± 514.30	During: 6.40 ± 0.95	Unclear	Unclear	50	High
					Swaddling	40	2–4	38.85 ± 1.00	3,369.75 ± 386.20	During: 5.85 ± 0.86	Unclear	Unclear	50	
					Swaddling + holding	40	2–4	39.00 ± 1.24	3,459.62 ± 476.88	During: 5.57 ± 1.23	Unclear	Unclear	50	
					Swaddling + holding + BM	40	2–4	39.32 ± 0.99	3,398.75 ± 538.40	During: 4.47 ± 1.19	Unclear	Unclear	50	
Pekyiğit (19)	2023	3	3	Türkiye	WN	30	2 ± 1.48	38–42	3,435 ± 408.33	Before: 0.00 ± 0.00	Unclear	Unclear	31.8	High
										During: 4 ± 0.74				
					FT	30	2.5 ± 0.74	38–42	3,130 ± 401.85	Before: 0.00 ± 0.00	Unclear	Unclear	34.1	
										During: 4 ± 0.74				
					WN + FT	30	2 ± 1.48	38–42	3,340 ± 385.19	Before: 0.00 ± 0.00	Unclear	Unclear	34.1	
										During: 2 ± 0.74				
Mir (20)	2018	3	3	Iran	YT	26	4.7 ± 1.0	38–42	2,500–3,990	After: 2.8 ± 1.6	Unclear	Unclear	53.8	High
					HW	26	5.1 ± 2.4	38–42	2,500–3,990	After: 4.8 ± 1.8	Unclear	Unclear	57.7	
					Control	26	4.1 ± 0.7	38–42	2,500–3,990	After: 4.4 ± 1.7	Unclear	Unclear	38.5	
Sapkota (21)	2021	2	2	Nepal	HW	46	1.17 ± 0.383	≧37	2,500–3,900	Total: 1.39 ± 0.57	Unclear	Unclear	37	Unclear
					Control	46	3.52 ± 3.371	≧37	2,500–3,900	Total: 2.20 ± 0.51	Unclear	Unclear	67.4	
Shu (22)	2014	3	3	Taiwan	Control	25	2.5 ± 1.8	38 ± 2.27	unclear	Before: 1.24 ± 1.36	7 72 ± 0.54	8 80 ± 0.5	48	High
										After: 4.64 ± 2.02				
					Swaddling	25	1.6 ± 1.1	38.5 ± 1.55	unclear	Before: 1.32 ± 1.49	7 88 ± 0.33	8 96 ± 0.2	68	
										After: 3.00 ± 2.74				
					HW	25	1.78 ± 1.9	38.51 ± 1.48	unclear	Before: 1.52 ± 1.26	7 64 ± 1.04	8 80 ± 0.58	44	
										After: 3.4 ± 2.22				
Orkisz (23)	2022	3	3	Poland	BM	30	>2	38–42	≥2,500	2.6 ± 2.5	>7	>7	Unclear	High
					ST	30	>2	38–42	≥2,500	3.1 ± 2.2	>7	>7	Unclear	
					NNS	30	>2	38–42	≥2,500	3.7 ± 2.6	>7	>7	Unclear	
Anbalagan (24)	2024	2	2	America	Control	46	1.96 ± 0.2	39.3 ± 1.23	Unclear	Before: 0 ± 1.85	Unclear	Unclear	48	Low
										During: 7 ± 0.74				
										1 min after: 5.5 ± 5.19				
										2 min after: 2 ± 5.19				
										3 min after: 0 ± 4.44				
										4 min after: 0 ± 4.44				
										5 min after: 0 ± 2.22				

					Music	54	2 ± 0.2	39.2 ± 1.09	Unclear	Before: 0 ± 0	Unclear	Unclear	57	
										During: 4 ± 4.44				
										1 min after: 0 ± 2.96				
										2 min after: 0 ± 2.22				
										3 min after: 0 ± 2.22				
										4 min after: 0 ± 0				
										5 min after: 0 ± 0				
Im (25)	2008	3	3	Korea	YT	33	<28	39.1 ± 1.36	3,184.2 ± 482.1	Before: 0.70 ± 1.29	8.5 ± 1.1	9.6 ± 0.5	45.45	High
										After: 4.15 ± 2.76				
					NNS	33	<28	39.1 ± 1.16	3,260.6 ± 512.3	Before: 0.39 ± 1.2	8.3 ± 0.8	9.5 ± 0.6	48.48	
										After: 4.06 ± 2.73				
					Control	33		38.9 ± 1.47	3,179.2 ± 394.8	Before: 0.88 ± 1.63	8.5 ± 1.1	9.4 ± 0.7	48.48	
										After: 4.79 ± 2.50				
Zhu (26)	2015	4	4	China	Control	61	3.11 ± 0.49	39.1 ± 1.26	3,140 ± 350	Before: 0.00 ± 0.01	>7	>7	51	High
										During: 6.43 ± 0.23				
										1 min after: 2.34 ± 0.29				
										5 min after: 0.74 ± 0.18				
					Music	62	3.37 ± 0.58	39.3 ± 1.63	3,280 ± 300	Before: 0.00 ± 0.02	>7	>7	52	
										During: 6.06 ± 0.22				
										1 min after: 1.98 ± 0.29				
										5 min after: 0.41 ± 0.17				
					BM	64	3.34 ± 0.48	39.6 ± 1.17	3,290 ± 410	Before: 0.00 ± 0.02	>7	>7	58	
										During: 3.08 ± 1.88				
										1 min after: 0.35 ± 0.27				
										5 min after: 0.09 ± 0.16				
					Music + BM	63	3.24 ± 0.5	39.5 ± 1	3,180 ± 450	Before: 0.00 ± 0.02	>7	>7	46	
										During: 4.38 ± 2.2				
										1 min after: 0.24 ± 0.28				
										5 min after: 0.01 ± 0.17				
Overgaard (27)	1999	2	2	Denmark	ST + disturbance^a^	49	6 ± 3.7	40 ± 3.28	3,550 ± 1,229	Before:0.00 ± 0.65	Unclear	Unclear	63.3	Low
										1 min after heel prick: 3 ± 5.19				
										1 min after blood sample: 0 ± 5.19				
					NNS + disturbance^a^	47	6 ± 2.96	40 ± 2.64	3,620 ± 1,029	Before: 0.00 ± 0.87	Unclear	Unclear	95.8	
										1 min after heel prick: 3 ± 5.19				
										1 min after blood sample: 0 ± 5.19				
Kadiroğlu (28)	2024	3	3	Türkiye	YT	22	4.82 ± 1.25	38.59 ± 0.85	3,276.59 ± 406.27	Before: 0.63 ± 0.72	Unclear	Unclear	54.5	Low
										During: 4.77 ± 1.79				
										After: 2.04 ± 1.17				
					wn	20	4.86 ± 1.12	38.77 ± 1.12	3,096.14 ± 305.14	Before: 0.45 ± 0.68	Unclear	Unclear	45.0	
										During: 5.45 ± 1.39				
										After: 2.80 ± 1.47				
					Control	22	4.55 ± 0.94	38.40 ± 0.94	3,195.80 ± 252.29	Befor: 0.72 ± 0.98	Unclear	Unclear	50	
										During: 5.59 ± 1.33				
										After: 3.72 ± 1.07				
Abbasoglu (29)	2,015	2	2	Türkiye	Acupressure + ST	21	4.9 ± 1.02	38.3 ± 0.84	3,247.6 ± 419.29	4.52 ± 0.87	8.6 ± 0.65	9.6 ± 0.65	42.8	High
					ST	21	4.9 ± 1.07	38.4 ± 0.87	3,420.0 ± 440.19	3.66 ± 1.01	8.4 ± 0.67	9.5 ± 0.59	66.6	
Gabriel (30)	2013	4	4	Spain	BM^a^	29	-	40 ± 3.7	2,266–4,338	During: 0 ± 0	6–9	8–10	68.6%	High
										1 min after: 1 ± 1.48				
										2 min after: 0 ± 0.74				
					st + ssc + touch + mv^a^	35	-	40 ± 2.96	2,340–4,108	During: 1 ± 0.74	5–10	8–10	48.6%	
										1 min after: 2 ± 1.48				
										2 min after: 0 ± 0.74				
					ssc + touch + mv^a^	31	-	39 ± 2.96	2,832–3,900	During: 0 ± 0.74	7–10	9–10	66.7%	
										1 min after: 4 ± 2.96				
										2 min after: 1 ± 1.48				
					st + touch + mv^a^	32	-	39 ± 2.96	1,945–4,176	During: 1 ± 0.74	7–10	9–10	66.7%	
										1 min after: 4 ± 2.22				
										2 min after: 1 ± 2.96				

Highlighted in red: selected for inclusion in the network analysis.

SD, standard deviation; BM, breastmilk; EBM, expressed breastmilk; SSC, skin-to-skin contact; FM, formula milk; ST, sweet taste; FT, facilitated tucking; KC, kangaroo care; MV, maternal voice; WN, white noise; HW, heel warming; VC, verbal comfort; YT, Yakson touch; WD, water drink; PS, placebo on the skin; NNS, non-nutritive sucking.

^a^ Redefined adjustments to the intervention after reading the full text.

### 2.4 Data analysis

In this study, we employed a Bayesian model for statistical analysis. The analysis was conducted using ADDIS 1.16.6 and the brms package in R statistical software (version 4.4.1). Mean differences (MD) and their 95% confidence intervals (CIs) were calculated to synthesize effect sizes.

A random-effects model was applied if the trials exhibited good homogeneity in study design, participants, interventions, controls, and outcomes. If significant heterogeneity was present in the pairwise meta-analysis (*I*² > 50%) or in the network meta-analysis (*τ*² > 0.1, or RESE > 1.5), further exploration of the heterogeneity source was conducted. Sensitivity and subgroup analyses were employed to investigate the sources of heterogeneity.

The node-splitting method was used for the inconsistency test. Outcome measures that did not form a network were excluded from the analysis. Initially, all arms were analyzed. However, since some arms were represented by only one or two studies, these arms were excluded in subsequent analyses to obtain a more stable network. If the second analysis resulted in significant improvements in RESD or other heterogeneity indicators, arms with fewer than three original studies were excluded. Funnel plots and Egger's test were used to assess publication bias in the relevant studies.

Study quality was assessed by RoB (Cochrane Collaboration's risk-of bias method) (31).

The methodology of this study evaluated several aspects of each trial, including random sequence generation and allocation concealment, blinding of participants, blinding of therapists, blinding of assessors, incomplete outcome data (attrition bias), and selective reporting (reporting bias). For the blinding of participants, we considered the risk of bias to be low, as infants are unable to self-report their pain. Each of these domains was rated as having a “low,” “high,” or “unclear” risk of bias (see Table 1). To assess the certainty of the evidence supporting the network estimates of the main outcomes, we used the Grading of Recommendations Assessment, Development, and Evaluation (GRADE) framework (32). To rank the interventions for each intervention, we used the surface under the cumulative ranking curve (SUCRA).

## 3 Results

### 3.1 Study selection

**Figure 1:**
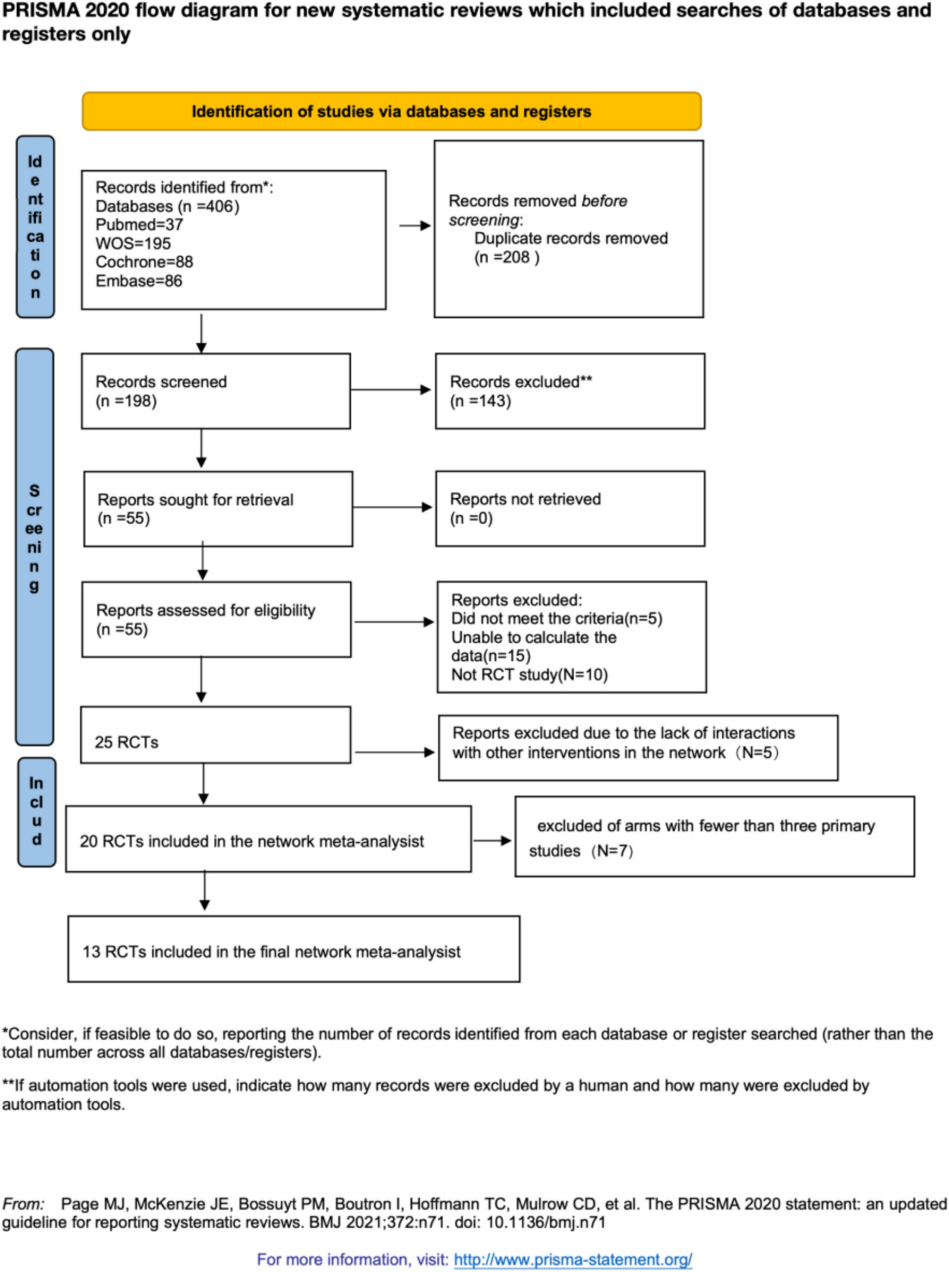
Study selection process.

We retrieved 406 studies from four databases. After removing duplicates using EndNote 21.4, 208 studies were identified as duplicates. A preliminary screening of the remaining 198 studies revealed that 143 did not meet the inclusion criteria. Following a full-text review of the remaining 55 studies, we identified 25 randomized controlled trials (RCTs) that met the inclusion criteria. However, 5 RCTs lacked interactions with other interventions in the network, leading to their exclusion. Ultimately, 20 RCTs were included in our network meta-analysis (NMA). Some intervention arms were represented by only one or two primary studies, making it challenging to conduct a robust network meta-analysis. Initially, we excluded arms with only one primary study, followed by exclusion of arms with two primary studies. By comparing the random-effects standard deviation (RESD) values, we found a significant reduction in RESD after excluding arms with two primary studies. Therefore, the results reported in this paper are based on the exclusion of arms with fewer than three primary studies (see Supplementary File S2). A flowchart of the study screening and selection process is provided in Figure 1.

### 3.2 Study characteristics

In total, 13 studies published between 2008 and 2024 were included in our network meta-analysis (NMA), involving 1141 neonates. Of these, 7 studies were two-arm trials, and 6 were three-arm trials. The studies focused on the following interventions: 5 studies on breast milking (BM), 3 studies on sweet taste (ST), 3 studies on non-nutritive sucking (NNS), 3 studies on Yakson touch (YT), 3 studies on swaddling, and 4 studies on heel warming (HW). In this study, the control group was defined as the no-intervention group, and we reclassified the interventions accordingly. Apaydin Cirik et al. (13), changing it from control to touch. Two studies provided incorrect definitions of the measures, so we redefined them (27, 30). Table 1 displays the general characteristics of the included studies.

### 3.3 Risk of bias of included studies

For random sequence generation, 9 studies reported the use of randomization schemes, including 3 studies that utilized envelopes, 4 studies that employed a computer-based random number table program, 1 study that applied a randomization block, and 1 study that used a coin for randomization. Four studies did not specify the randomization method employed (10, 11, 21, 22). We decided to include the four studies that did not specify a randomization method in our analysis, as excluding them resulted in only a minor reduction in the *τ*² value from 2.22 to 1.36, with no significant differences observed in other metrics such as effect sizes and I². Moreover, excluding these studies led to a widening of the 95% confidence intervals for the random-effects standard deviation (RSED) and inconsistency standard deviation (ISD) (see Supplementary File S3). Therefore, we concluded that these studies did not substantially impact the heterogeneity, consistency, or transitivity of our findings.

**Figure 2:**
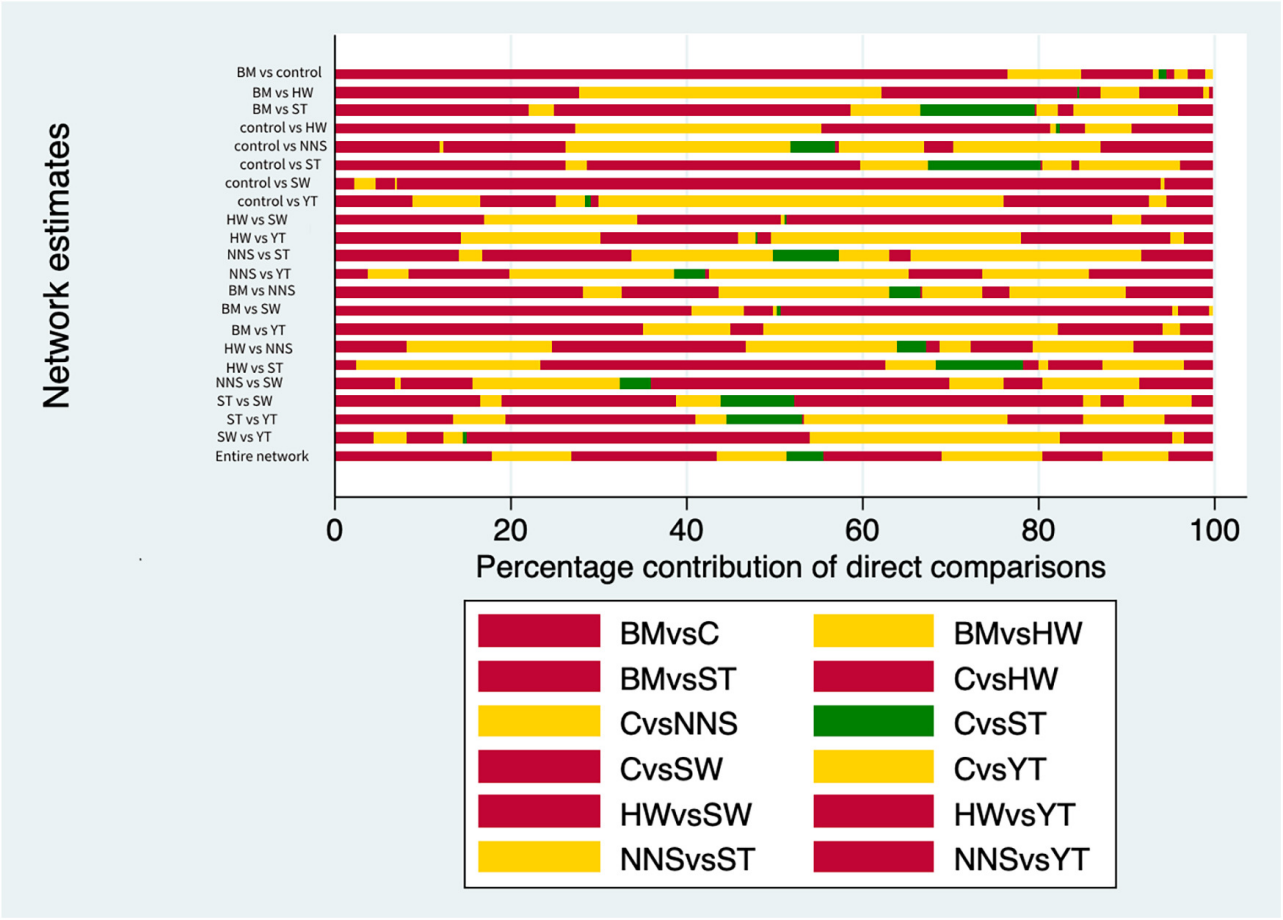
The risk of bias charts for the arms. C, control; BM, breastmilk; ST, sweet taste; NNS, nonnutritive sucking; HW, heel warming.

In terms of baseline balance, all 13 studies demonstrated balance at baseline. However, many interventions, such as breastfeeding, non-nutritive sucking (NNS), and swaddling, cannot be blinded during assessment, leading to potential bias in the blinding process across the studies. Regarding the risk of bias due to missing outcome data, all studies included established outcome data. In total, 2 studies were classified as low risk, 9 studies as high risk, and 2 studies as unclear. The risk of bias charts for the arms are shown in Figure 2.

#### 3.4.1 Pairwise meta-analysis

We conducted a pairwise meta-analysis of clinical effectiveness, which is presented in Supplementary File S4. The results showed that for the control group, breast milking (BM) vs. control (mean: 2.3, CI: 2.09–2.51, *I*² = 35.7%), swaddling vs. control (mean: 0.66, CI: 0.29–1.03, *I*² = 13%), and Yakson touch (YT) vs. control (mean: 1.25, CI: 0.68–1.83, *I*² = 0.0%) were all effective, with the most effective intervention being BM vs. control.

For comparisons between interventions, sweet taste (ST) vs. non-nutritive sucking (NNS) (mean: 1.13, CI: 0.01–2.25, *I*² = 33.4%) and BM vs. ST (mean: 0.78, CI: 0.07–1.50, *I*² = 0%) were effective. However, heel warming (HW) vs. control (mean: 0.5, CI: 0–1.03, *I*² = 72.6%) and NNS vs. control (mean: −0.36, CI: −1.45–0.74, *I*² = 20.9%) were not found to be effective.

All studies exhibited low heterogeneity, except for HW vs. control, which showed significant heterogeneity (*I*² = 72.6%). We performed subgroup and sensitivity analyses for the HW vs. control group but did not identify the source of the heterogeneity. The results of these analyses are provided in Supplementary File S5.

#### 3.4.2 Network meta-analysis

**Figure 3:**
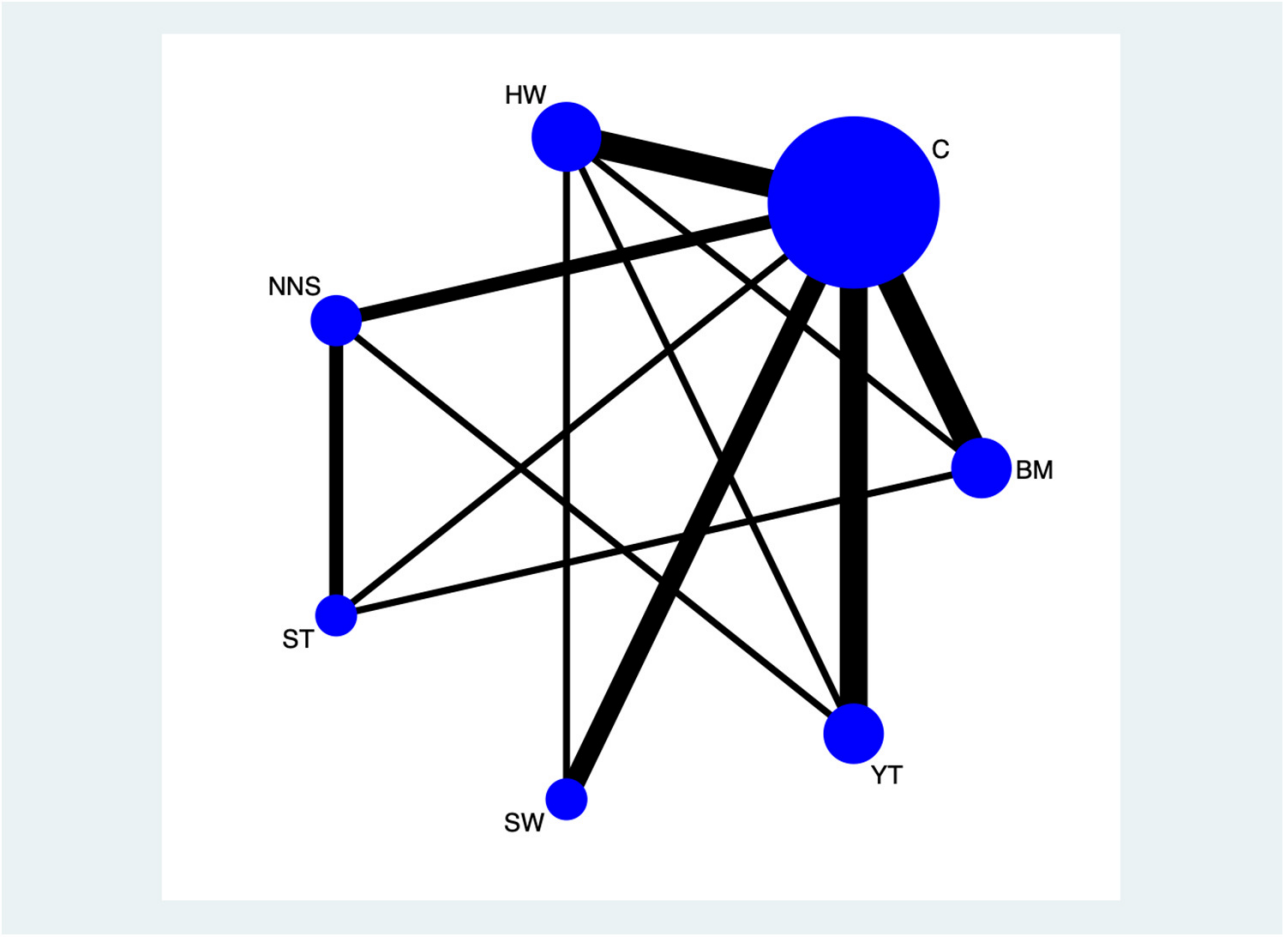
Network of direct comparison for the interventions of non-pharmacological pain management. Each node represents 1 intervention. The size of the node is proportional to the number of participants randomized to that intervention. The edges represent direct comparisons, and the width of the edge is proportional to the number of trials. C, control; BM, breastmilk; ST, sweet taste; NNS, nonnutritive sucking; HW, heel warming.

**Figure 4:**
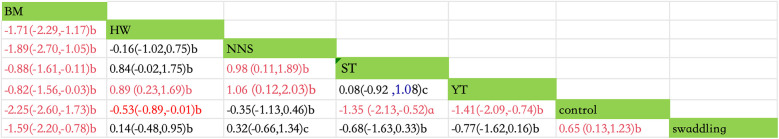
Comparisons for efficacy of the interventions. Data are mean (95% CrI) in the column-defining intervention compared with the row-defining intervention. Valid outcomes are highlighted in red. The certainty of the evidence (according to GRADE) was incorporated in this Figure. CrI, credible interval; C, control; BM, breastmilk; ST, sweet taste; NNS, nonnutritive sucking; HW, heel warming; **(a)** moderate level of GRADE; **(b)** low Level of GRADE; **(c)** very low Level of GRADE.

Figure 2 illustrates the network diagram of the clinical effectiveness of non-pharmacological interventions. The Bayesian command lines, and the degree of aggregation are presented in Supplementary File S3, from which it is evident that the model converges well. Figure 3 showed the network of direct comparison for the interventions of non-pharmacological pain management. Figure 4 displays the comparative effects of mean differences (MDs) and corresponding 95% confidence intervals (CIs) for different interventions in detail. From Figure 4, we observe that BM vs. control (MDs: −2.25, 95% CI: −2.60, −1.73), BM vs. ST (MDs: −0.88, 95% CI: −1.61, −0.11), BM vs. YT (MDs: −0.82, 95% CI: −1.56, −0.03), BM vs. NNS (MDs: −1.89, 95% CI: −2.70, −1.05), BM vs. swaddling (MDs: −1.59, 95% CI: −2.20, −0.78), and BM vs. HW (MDs: −1.71, 95% CI: −2.29, −1.17) were all effective. Additionally, HW vs. YT (MDs: 0.89, 95% CI: 0.23, 1.69), NNS vs. YT (MDs: 1.06, 95% CI: 0.12, 2.03), YT vs. control (MDs: −1.51, 95% CI: −2.09, −0.74), NNS vs. ST (MDs: 0.98, 95% CI: 0.11, 1.89), ST vs. control (MDs: −1.35, 95% CI: −2.13, −0.52), control vs. swaddling (MDs: 0.65, 95% CI: 0.13, 1.23), and HW vs. control (MDs: −0.53, 95% CI: −0.89, −0.01) were also effective. However, NNS vs. control (MDs: −0.35, 95% CI: −1.13, 0.46) was not effective compared to the control group.

**Figure 5:**
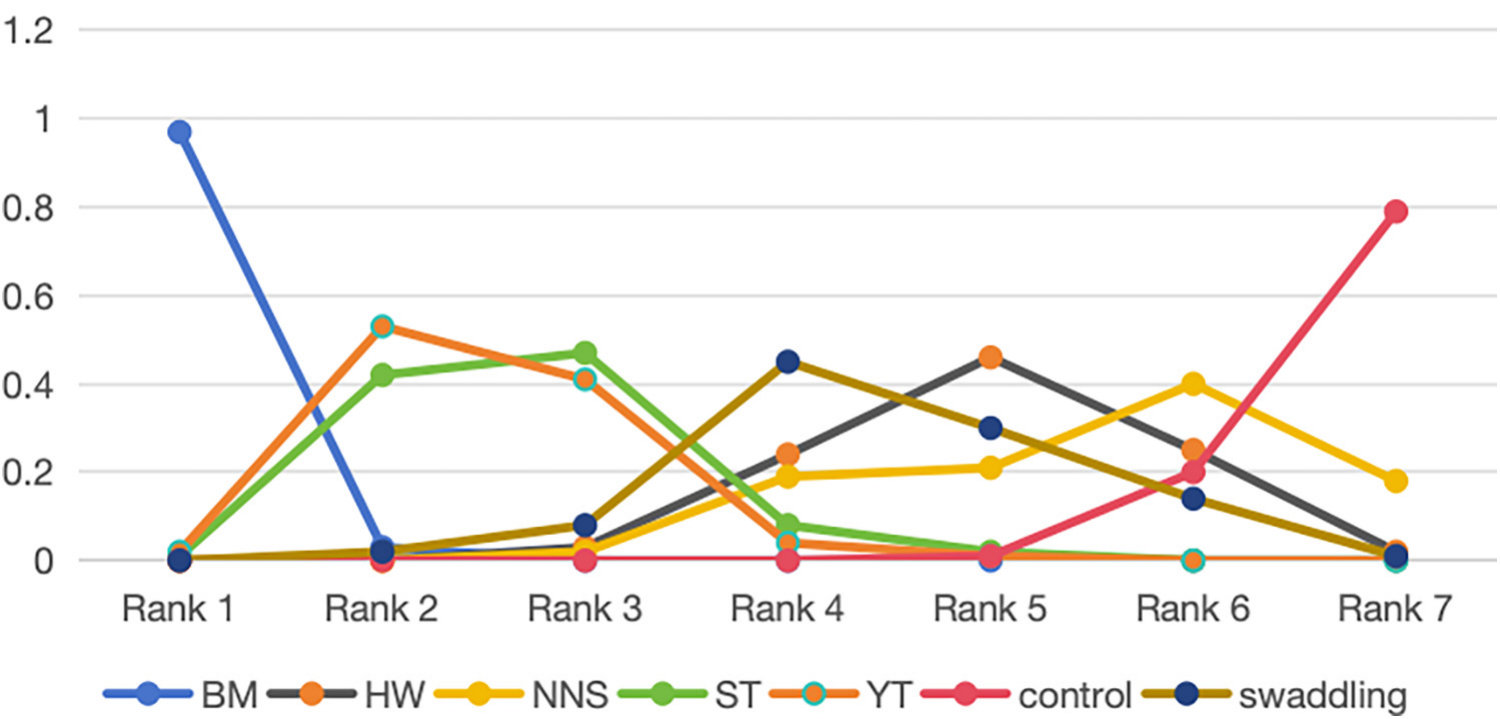
SUCRAs of all trials for efficacy. BM, breastmilk; ST, sweet taste; NNS, nonnutritive sucking; HW, heel warming.

The numerical results of the Surface Under the Cumulative Ranking Curve (SUCRA) also revealed that BM is the best non-pharmacological pain management for neonates, as shown in Supplementary File S6. The SUCRA values for specific intervention measures were ranked as follows: BM (SUCRA: 97% for rank 1) > YT (SUCRA: 53% for rank 2) > ST (SUCRA: 47% for rank 3) > swaddling (SUCRA: 45% for rank 4) > HW (SUCRA: 46% for rank 5) > NNS (SUCRA: 40% for rank 6) > control (SUCRA: 79% for rank 7). Additionally, the cumulative probability of the ranking results for all interventions was calculated using ADDIS 1.16.6, as shown in Figure 5.

In terms of consistency, node-splitting analysis, inconsistency standard deviation (ISD), and inconsistency factors (IF) were used to assess the consistency of certain comparison results using ADDIS 1.16.6. All *p*-values from the node-splitting analysis were greater than 0.05, indicating no significant inconsistency. The ISD was 0.36 (95% CrI: 0.01, 1.36), which is considered low, and the confidence intervals of the IF for all comparisons spanned zero. These results suggest that there was no statistical inconsistency, and the models exhibited good consistency. All findings are presented in Supplementary File S7.

In terms of heterogeneity, random effects standard deviation (RESD) analyzed using ADDIS 1.16.6, along with *τ*² and *I*² analyzed via the brms package in R (version 4.4.1), were employed to test the heterogeneity of comparison results. The RESD in the consistent model was 0.28 (95% CrI: 0.04, 0.73), while the RESD in the inconsistent model was 0.36 (95% CrI: 0.01, 1.36). The *I*² value was 100%, and *τ*² was 2.22, indicating high heterogeneity. To explore the sources of heterogeneity, meta-regression, sensitivity analysis, subgroup analysis, and publication bias tests were conducted.

In the meta-regression analysis, we identified several significant factors influencing neonatal pain relief outcomes. A positive association was found between average age and pain relief, while average weight, publication year, and some countries showed a negative association with pain relief outcomes. Subgroup analyses were performed based on baseline pain scores, risk of bias (ROB) scores, study location, country, publication year, and trial design (two-arm or multi-arm). Sensitivity analyses were also conducted by excluding studies with incomplete reporting, those with small sample sizes, and sequentially removing individual studies to explore potential sources of heterogeneity. These results are detailed in Supplementary File S8. When Mir 2018 was excluded, the RESD and *τ*² values showed a noticeable reduction, from 0.28 (95% CrI: 0.04, 0.73) to 0.18 (95% CrI: 0.01, 0.59) for RESD, and from 2.22 to 1.55 for *τ*². However, *I*² remained at 100%, and no significant decrease in heterogeneity was observed in other analyses. This may be due to the small sample size, which led to instability in the heterogeneity estimates.

**Figure 6:**
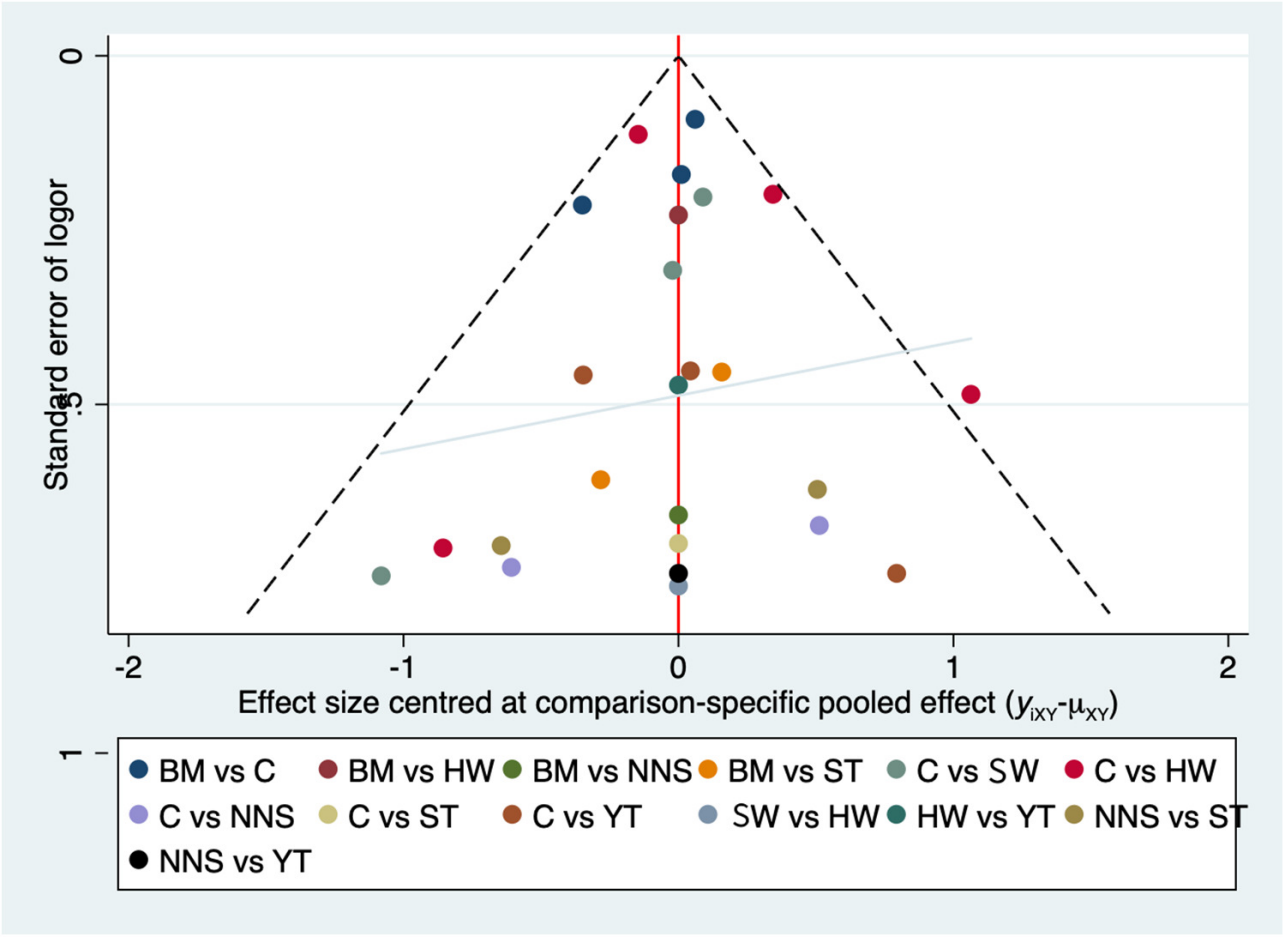
The publication bias chart for clinical effectiveness. BM, breastmilk; ST, sweet taste; NNS, nonnutritive sucking; HW, heel warming; SW, swaddling; C, control.

The publication bias chart for clinical effectiveness can be seen in Figure 6. The funnel plot showed a symmetric distribution of scatter points, with an equal number on both the left and right sides. We also performed Egger's linear regression in R, which yielded *p* = 0.1322, *b* = 4.9118 (95% CI: 3.7745, 6.0490). The non-zero intercept of the regression indicates potential systematic bias. To address this, we conducted the Trim-and-Fill method, which indicated an estimated four missing studies on the right side (SE = 3), suggesting the presence of publication bias (see Supplementary File S9). So, one possible explanation for the high heterogeneity was the influence of publication bias.

In terms of transitivity, our study focused on healthy newborns who did not experience asphyxia, ensuring consistent baseline characteristics across studies. Additionally, heel-stick blood sampling is a highly standardized procedure with minimal operator variability. All included studies were randomized controlled trials (RCTs) with similar study designs, further supporting the consistency of our analysis. The RESD in the consistency model was 0.28 (95% CrI: 0.04, 0.73), while the RESD in the inconsistency model was 0.24 (95% CrI: 0.02, 0.72), the minimal differences between the data indicate strong transitivity. Sensitivity analyses were performed by sequentially removing one arm at a time, and the main results, including the RESD values, remained unchanged. These results are provided in Supplementary File S10. Furthermore, node-splitting tests showed no statistical significance, further supporting the robustness of the transitivity assumption. Overall, the evidence suggests that transitivity was strong in our analysis.

### 3.5 Confidence in evidence

We used the GRADE framework to assess the clinical effectiveness of each intervention. The results are presented in Supplementary File S11. Based on the evaluation, the quality of evidence was categorized as “high,” “moderate,” “low,” or “very low.” Additionally, we employed contribution plots to identify the direct comparisons that most influenced the results of the network meta-analysis (see Supplementary File S12). The evaluation results indicated moderate quality for the ST vs. control group, very low quality for the NNS vs. swaddling group, and very low quality for the ST vs. YT group. For the remaining groups, the quality of evidence was low. As illustrated in the contribution plots in Supplementary File S12, the direct comparisons of ST vs. control, HW vs. swaddling, and NNS vs. YT contributed 12.9%, 4.5%, and 15.4% to the entire network, respectively. Given these contributions, the results should be interpreted with caution.

## 4 Discussion

To expand the evidence base on non-pharmacological interventions for pain management in neonates, we conducted the first network meta-analysis (NMA) evaluating the effectiveness of these interventions, drawing on recent RCTs. This study included data from 13 RCTs, comprising 981 neonates who were randomly assigned to either pain management or control groups.

Both the pairwise meta-analysis and the SUCRA values suggest that breastfeeding (BM) may offer superior clinical effectiveness for pain management during procedures. BM has been previously reported as effective in alleviating pain associated with minor painful procedures in newborns (17). BM is a comprehensive intervention that encompasses skin-to-skin contact, mother-infant communication, stimulation of peripheral sensory receptors, and activation of the sense of taste, all of which contribute to its remarkable analgesic effects.

Yakson touch (YT) ranked as the second most effective intervention, showing effectiveness in the pairwise meta-analysis, particularly in the YT vs. control group comparison. Yakson, meaning “healing hand,” refers to the natural and nurturing action in which a mother places her hand on her child's painful area and gently caresses or massages it to alleviate discomfort. This method can be considered one of the most basic and instinctive forms of therapy (25). Since Yakson touch involves energy transfer, we initially excluded it from the analysis due to concerns about its potential impact on transitivity. However, after conducting a sensitivity analysis, we found that Yakson touch did not affect the primary results. Consequently, we decided to include it in the study.

Sweet taste (ST) was ranked as the third most effective intervention and demonstrated effectiveness in the pairwise meta-analysis, particularly in the ST vs. control group comparison. In this study, we combined dextrose, glucose, and sucrose interventions without regard to their density or dose, due to the limited number of studies available. The mechanism behind the use of oral glucose water for neonatal pain relief is believed to involve both physiological and neurobiological processes. Oral sweet liquids have been shown to activate the brain's reward system, leading to the release of endogenous opioids, such as endorphins, which help reduce pain perception. Additionally, glucose may trigger a calming effect by stimulating the release of serotonin, a neurotransmitter that contributes to relaxation and pain relief. This combined effect helps alleviate discomfort in neonates during painful procedures, making it a simple and effective analgesic option in clinical settings.

Swaddling was ranked as the fourth most effective intervention and demonstrated effectiveness in the pairwise meta-analysis, particularly in the swaddling vs. control group comparison. Swaddling is believed to enhance the self-regulation abilities of neonates by promoting calmness, improving sleep, and soothing crying. Theories suggest that swaddling alleviates pain through sensory or multisensory stimulation, which may contribute to its analgesic effect. Evidence has shown that swaddling can be particularly effective in reducing pain during heel stick procedures, providing a simple and non-invasive intervention for neonatal pain management (33). Safe swaddling, which involves keeping the legs close to the body in a flexed position, in alignment with the neonate's natural anatomy, has been shown to provide various positive effects. These include promoting comfort and stability in neonates.

Heel warming (HW) and non-nutritive sucking (NNS) were ranked as the fifth and sixth most effective interventions, respectively. However, they were not found to be effective in the pairwise meta-analysis, particularly in the HW vs. control group and NNS vs. control group comparisons.

## 5 Limitations

Our review has several limitations. Although the RESD indicated minimal within-group heterogeneity, the *τ*² and *I*² values suggested extremely high between-group heterogeneity. One potential source of this heterogeneity was the small number of studies available, which led us to combine dextrose, glucose, and sucrose interventions without considering variations in concentration or dosage. These factors may have contributed to the observed heterogeneity.

The meta-regression results highlight the complexity of neonatal pain relief, emphasizing the influence of biological factors (such as age and weight), cultural factors (such as country of origin), and procedural factors (such as intervention methods). Tailoring interventions to account for these variables could improve pain management outcomes for neonates. Future research should investigate the mechanisms underlying these associations to refine clinical protocols and reduce disparities in care.

According to the GRADE framework, the quality of many comparisons was assessed as low or very low. A significant limitation was that many trials did not ensure proper concealment for assessment, particularly in interventions such as breastfeeding, swaddling, and non-nutritive sucking, which limits the interpretation of these findings. We focused only on average intervention effects and were unable to explore potentially important clinical and demographic factors that might modify the intervention response at the individual patient level. Furthermore, due to limited reporting in the original studies, we could not quantify certain outcomes, such as the potential influence of race on intervention effects. The number of publications included in our analysis was also influenced by the strict inclusion criteria, as we focused specifically on heel-stick procedures assessed by the NIPS scale in neonates and restricted our review to RCTs. As a result, several relevant studies that did not meet the inclusion criteria may have been missed.

## 6 Conclusions

Due to the limitation of high heterogeneity, this study can be defined as a clinical effectiveness comparison. This network meta-analysis offers a comprehensive comparison of interventions; however, it is important to acknowledge the high level of heterogeneity observed across the included studies. This variability may stem from differences in study design, patient characteristics, publication year, country of origin, and specific interventions. While these factors may limit the generalizability of the findings, our analysis still provides valuable insights into the effectiveness of various interventions compared to no intervention, specifically breastfeeding (BM), sweet taste (ST), Yakson touch (YT), and swaddling. Given the observed variability, we recommend exercising caution when interpreting the results, particularly in the ranking of SUCRA, as the effectiveness of the interventions may vary across different subpopulations or settings.

The findings underscore the need for further research with more homogeneous study populations and standardized methodologies to better understand the true effects of non-pharmacological interventions. This study contributes to the existing body of evidence by highlighting areas of uncertainty and identifying potential sources of variability within the intervention landscape. While the high heterogeneity limits the robustness of some conclusions, the paper offers important directions for future research, including the need for more focused and methodologically consistent trials.

## Data Availability

The original contributions presented in the study are included in the article/Supplementary Material, further inquiries can be directed to the corresponding author.
