# Supplementary material for: A Bayesian network meta-analysis of non-pharmacological interventions for neonatal pain management: a clinical effectiveness comparison
Source: Front Pediatr. 2025 May 22;13:1547308. doi: 10.3389/fped.2025.1547308 (PMC12137100; doi:10.3389/fped.2025.1547308)

***Supplementary Material***

***The effectiveness of non-pharmaceutical interventions to improve pain for newborn, a network meta-analysis***

Lingxue Xu^1^, Lali Xiang^1^, Bo Zheng^1^, Lihui Pan^1^, Peipei Xue^1^, Juan Li^1^, Yurong He^1^, Hongyan Liu^1^, Yuwei Hu^1^, Bo Zheng^2^*

**Affiliations: 1**. Yuhuan People’s Hospital, Taizhou, China

2. UNC of Greensboro, NVC USA

**Address correspondence to**: Bo Zheng, UNCG, NC, USA (e-mail:dydra361@gmail.com)

**Supplementary file 1**

the formula we used

we would combined the data of baseline, during the procedure, and after the procedure. we combine the mean with the formula: mean=(mean_1_​+mean_2_​+mean_3_......+mean_n_−mean_0_) /n

SD= √(SD_0_^2^+SD_1_^2^+SD_2_^2^+......SD_N_^2^)/N

Mean0 and SD0 represents the baseline data of mean and sd prior to the operation. In calculating standard deviation (SD), our primary focus is on the variation in data observed during the experiment rather than the differences between experimental values and the baseline, so We opted to use the formula SD= √(SD_0_^2^+SD_1_^2^+SD_2_^2^+......SD_N_^2^)/N, rather than rely on SD= √(SD_0_^2^+SD_1_^2^+SD_2_^2^+......SD_N_^2^)/(N+1).

**Supplementary file 2**

the RESD for network with two primary studies of arms.

| Network | PSRF | iterations | RESD  mean+95%CrI |
| --- | --- | --- | --- |
| excluded with one primary studies of arms | 1.00 | number of chains:4  tuning iterations:20000  simulation iterations:5000  thinning interval:10  inference samples:10000  variance scaling factor:2.5 | 0.64 (0.32, 1.15) |
| excluded with two primary studies of arms | 1.00 | number of chains:4  tuning iterations:20000  simulation iterations:5000  thinning interval:10  inference samples:10000  variance scaling factor:2.5 | 0.28 (0.04,0.73) |

PSRF: Potential Scale Reduction Factor; RESD: Random Effects Standard Deviation

**Supplementary file 3**

the result for network compares all 13 RCTs with 9 RCTs (introduced the randomization schemes)

the original data were provided in dataset 1.

the code for r to analyze I2 and τ^2^:

model <- brm(

formula = effect | se(se) ~ trt + (1|study),

data = data,

family = gaussian(),

prior = c(

prior(normal(0, 5), class = "b"),

prior(normal(0, 5), class = "Intercept"),

prior(normal(0, 5), class = "sd") #tau

),

chains = 4, # MCMC

iter = 4000, #

warmup = 2000, # warm

control = list(adapt_delta = 0.99, max_treedepth = 20)

)

| Network | PSRF | iterations | RESD  mean  （95%CrI） | τ^2^ | I^2^ (%) | ISD  mean（95%） | rank |
| --- | --- | --- | --- | --- | --- | --- | --- |
| 13 RCTs | 1.00 | number of chains:4  tuning iterations:20000  simulation iterations:5000  thinning interval:10  inference samples:10000  variance scaling factor:2.5 | 0.28 (0.04, 0.73) | 2.22 | 100 | 0.36 （0.01，1.36） | BM>YT>ST>swaddling>HW>NNS>control |
| 9 RCTs | 1.00 | number of chains:4  tuning iterations:20000  simulation iterations:200000  thinning interval:10  inference samples:10000  variance scaling factor:2.5 | 0.23 (0.02, 0.84) | 1.36 | 100 | 0.49 (0.04,1.90) | BM>YT>ST>NNS>swaddling>HW>control |

PSRF: Potential Scale Reduction Factor; RESD: Random Effects Standard Deviation；ISD: Inconsistency Standard Deviation; BM=breastmilk, ST=sweet taste, HW=heel warming, YT=Yakson touch, NNS=non-nutritive sucking.

We used ADDIS 1.16.6 and brms package in the R statistical software (version 4.4.1) for this analysis. Apart from τ2 , no other metrics showed a significant reduction.

**Supplementary file 4**

Pairwise meta-analysis


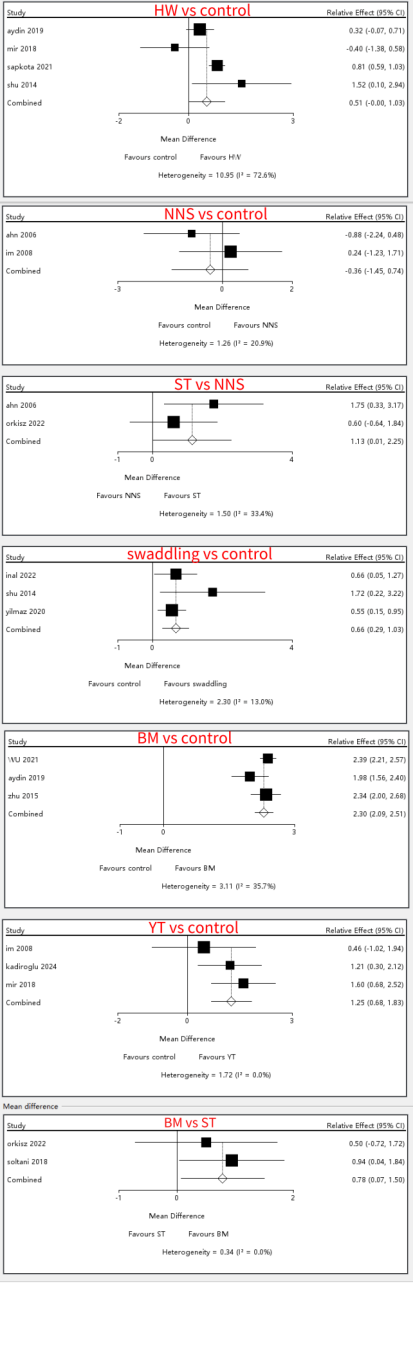


BM=breastmilk, ST=sweet taste, HW=heel warming,

YT=Yakson touch, NNS=non-nutritive sucking.

**Supplementary file 5**

subgroup and sensitivity analysis for Pairwise meta-analysis of HW vs control

| HW vs control | mean (95 CrI) | I2(%) |
| --- | --- | --- |
| subgroup by risk stratification | | |
| unclear risk | 0.59 (0.12,1.07) | 78.3% |
| high risk | 0.49 (-1.39, 2.37) | 80.1% |
| sensitivity analysis through stepwise exclusion | | |
| excluded mir 2018 | 0.68 (0.22, 1.63) | 66.4% |
| excluded aydin 2019 | 0.59 (-0.30, 1.48) | 71.7% |
| excluded sapkota 2021 | 0.34 (-0.44, 1.13) | 60.6% |
| excluded shu 2014 | 0.39 (-0.15, 0.94) | 78.8% |

**Supplementary file 6**

surface under the cumulative ranking curve

| intervention | Rank 1 | Rank 2 | Rank 3 | Rank 4 | Rank 5 | Rank 6 | Rank 7 |
| --- | --- | --- | --- | --- | --- | --- | --- |
| BM | 0.97 | 0.03 | 0 | 0 | 0 | 0 | 0 |
| HW | 0 | 0 | 0.03 | 0.24 | 0.46 | 0.25 | 0.02 |
| NNS | 0 | 0 | 0.02 | 0.19 | 0.21 | 0.4 | 0.18 |
| ST | 0.01 | 0.42 | 0.47 | 0.08 | 0.02 | 0 | 0 |
| YT | 0.02 | 0.53 | 0.41 | 0.04 | 0.01 | 0 | 0 |
| control | 0 | 0 | 0 | 0 | 0.01 | 0.2 | 0.79 |
| swaddling | 0 | 0.02 | 0.08 | 0.45 | 0.3 | 0.14 | 0.01 |

BM=breastmilk, ST=sweet taste, HW=heel warming,

YT=Yakson touch, NNS=non-nutritive sucking.

**Supplementary file 7**

Node splitting analysis

| Name | Direct Effect | Indirect Effect | Overall | P-Value |
| --- | --- | --- | --- | --- |
| BM, HW | 1.75 (0.73, 2.64) | 1.66 (0.94, 2.51) | 1.71 (1.17, 2.29) | 0.86 |
| BM, NNS | 1.18 (-0.23, 2.56) | 2.26 (1.21, 3.26) | 1.89 (1.05, 2.70) | 0.2 |
| BM, ST | 0.88 (-0.01, 1.76) | 0.72 (-0.73, 2.22) | 0.88 (0.11, 1.61) | 0.86 |
| BM, control | 2.26 (1.70, 2.78) | 2.09 (1.03, 2.86) | 2.25 (1.73, 2.60) | 0.73 |
| HW, YT | -1.58 (-2.73, -0.62) | -0.39 (-1.32, 0.56) | -0.89 (-1.69, -0.23) | 0.06 |
| HW, swaddling | -0.65 (-2.15, 0.96) | -0.04 (-0.92, 0.65) | -0.14 (-0.95, 0.48) | 0.48 |
| NNS, ST | -1.08 (-2.09, -0.05) | -0.96 (-2.39, 0.56) | -0.98 (-1.89, -0.11) | 0.89 |
| NNS, YT | -0.29 (-1.71, 1.21) | -1.52 (-2.64, -0.39) | -1.06 (-2.03, -0.12) | 0.14 |
| NNS, control | -0.26 (-1.28, 0.74) | 0.86 (-0.12, 1.83) | 0.35 (-0.46, 1.13) | 0.07 |
| ST, control | 0.50 (-0.88, 1.94) | 1.63 (0.79, 2.49) | 1.35 (0.52, 2.13) | 0.16 |

BM=breastmilk, ST=sweet taste, HW=heel warming, YT=Yakson touch, NNS=non-nutritive sucking.

IF (Inconsistency Factors)

| cycle | Median (95%CrI) |
| --- | --- |
| BM, HW, NNS, YT | 0.08 (-0.56, 1.16) |
| HW, NNS, ST, YT, control, swaddling | 0.01 (-0.90, 0.85) |
| BM, HW, NNS, ST, YT, control | -0.1 (-1.16, 0.37) |
| HW, NNS, ST, YT, control | -0.26 (-1.39, 0.21) |
| NNS, ST, YT, control | 0.00 (-0.92, 0.76) |
| BM, HW, NNS, ST, YT | -0.02 (-1.07, 0.72) |
| NNS, YT, control | 0.04 (-0.78, 0.97) |

BM=breastmilk, ST=sweet taste, HW=heel warming,

YT=Yakson touch, NNS=non-nutritive sucking.

**Supplementary file 8**

meta-regression analysis

Subsequently, we employed mean imputation to address the missing values.

the original data is provided in dataset 2.

| **Variable** | **Estimate** | **SE** | **z-value** | **p-value** | **95% CI (Lower, Upper)** | **Significance** |
| --- | --- | --- | --- | --- | --- | --- |
| **Intercept** | 1995.9173 | 608.8533 | 3.2782 | 0.001 | (802.5868, 3189.2478) | ** |
| **Age Mean** | 0.6634 | 0.2309 | 2.8726 | 0.0041 | (0.2107, 1.1160) | ** |
| **Age SD** | -0.1191 | 0.4337 | -0.2747 | 0.7835 | (-0.9693, 0.7310) | Not significant |
| **ROB: Unclear** | -1.1505 | 0.616 | -1.8678 | 0.0618 | (-2.3577, 0.0568) | . |
| **ROB: High** | -0.3133 | 0.4356 | -0.7193 | 0.472 | (-1.1669, 0.5404) | Not significant |
| **Weight Mean** | -0.0083 | 0.0026 | -3.1416 | 0.0017 | (-0.0135, -0.0031) | ** |
| **Weight SD** | -0.0031 | 0.0024 | -1.3168 | 0.1879 | (-0.0078, 0.0015) | Not significant |
| **Country: Iran** | 3.9745 | 1.3393 | 2.9677 | 0.003 | (1.3496, 6.5994) | ** |
| **Country: Korea** | -7.4504 | 1.771 | -4.2068 | < 0.0001 | (-10.9215, -3.9792) | *** |
| **Country: Nepal** | 5.482 | 3.0151 | 1.8182 | 0.069 | (-0.4274, 11.3914) | . |
| **Country: Poland** | 6.3226 | 3.1562 | 2.0033 | 0.0452 | (0.1366, 12.5086) | * |
| **Country: Taiwan** | -0.5127 | 1.2095 | -0.4239 | 0.6717 | (-2.8831, 1.8578) | Not significant |
| **Country: Turkey** | 7.876 | 2.4591 | 3.2029 | 0.0014 | (3.0563, 12.6956) | ** |
| **Year** | -0.9756 | 0.2994 | -3.2584 | 0.0011 | (-1.5624, -0.3887) | ** |
| data deficency | -0.0214 | 0.7128 | -0.0301 | 0.976 | (-1.4185, 1.3756) | Not significant |
| **Treatment: feed（breastmilk）** | -1.1929 | 0.456 | -2.6157 | 0.0089 | (-2.0867, -0.2990) | ** |
| **Treatment: HW** | -0.0601 | 0.4267 | -0.1409 | 0.8879 | (-0.8964, 0.7761) | Not significant |
| **Treatment: NNS** | 0.8426 | 0.625 | 1.3481 | 0.1776 | (-0.3824, 2.0676) | Not significant |
| **Treatment: ST** | 0.1873 | 0.6624 | 0.2828 | 0.7773 | (-1.1109, 1.4856) | Not significant |
| **Treatment: SW** | -0.1742 | 0.4093 | -0.4255 | 0.6704 | (-0.9764, 0.6281) | Not significant |
| **Treatment: YT** | -0.1858 | 0.5687 | -0.3267 | 0.7439 | (-1.3005, 0.9289) | Not significant |
| **Male** | -0.0124 | 0.0203 | -0.6115 | 0.5409 | (-0.0521, 0.0273) | Not significant |

the code for r to analyze I2 and τ^2^ in R :

model <- brm(

formula = effect | se(se) ~ trt + (1|study),

data = data,

family = gaussian(),

prior = c(

prior(normal(0, 5), class = "b"),

prior(normal(0, 5), class = "Intercept"),

prior(normal(0, 5), class = "sd") #tau

),

chains = 4, # MCMC

iter = 4000, #

warmup = 2000, # warm

control = list(adapt_delta = 0.99, max_treedepth = 20)

)

subgroup analysis and sensitivity analysis

|  | RESD  mean（95%CrI） | τ^2^ | I^2^ (%) |
| --- | --- | --- | --- |
| subgroup by country | | | |
| turkey | 0.54(0.02, 1.86) | 2.07 | 100 |
| not turkey | 0.47 (0.11,1.24) | 1.36 | 100 |
| subgroup by baseline | | | |
| have baseline data | 0.24 (0.01, 1.64) | 2.26 | 100 |
| don’t have baseline data | 0.47 (0.08, 1.47) | 1.89 | 100 |
| sensitivity analysis | | | |
| for studies with big sample size(>50) | 0.29 (0.03, 0.79) | 1.57 | 100 |
| for studies in high level ROB | 0.28 (0.01, 1.01) | 1.59 | 100 |
| for studies that have comprehensive data (included data for during and after procedure) | 0.40 (0.02, 2.20) | 2.07 | 100 |
| excluded Korea | 0.24 (0.03, .073) | 2.82 | 100 |
| excluded the study published in 2024 | 0.50 (0.10, 1.46) | 2.82 | 100 |
| for studies had multiply arms | 0.45 (0.05, 1.34) | 1.2 | 100 |
| sensitivity analysis by exluding study one by one | | | |
| excluded Soltani 2018 | 0.30 (0.06, 0.82) | 1.44 | 100 |
| excluded wu 2021 | 0.34 (0.07, 0.86) | 1.47 | 100 |
| excluded ahn 2006 | 0.25 (0.04, 0.69) | 1.57 | 100 |
| excluded inal 2022 | 0.35 (0.06, 0.89) | 1.58 | 100 |
| excluded aydin 2019 | 0.32 (0.04, 0.94) | 1.42 | 100 |
| excluded yilmaz 2020 | 0.32 (0.07, 0.84) | 1.36 | 100 |
| excluded Mir 2018 | 0.18 (0.01, 0.59) | 1.55 | 100 |
| excluded Sapkota 2021 | 0.21 (0.01, 0.72) | 1.43 | 100 |
| excluded shu 2914 | 0.30 (0.06, 0.78) | 1.52 | 100 |
| excluded Orkisz 2022 | 0.28 (0.02, 0.79) | 1.56 | 100 |
| excluded im 2008 | 0.26 (0.04, 0.72) | 1.57 | 100 |
| excluded zhu 2015 | 0.35 (0.05, 0.87) | 1.58 | 100 |
| excluded Kadiroğlu 2024 | 0.29 (0.04, 0.80) | 1.55 | 100 |

**Supplementary file 9**

trim-and-fill method.


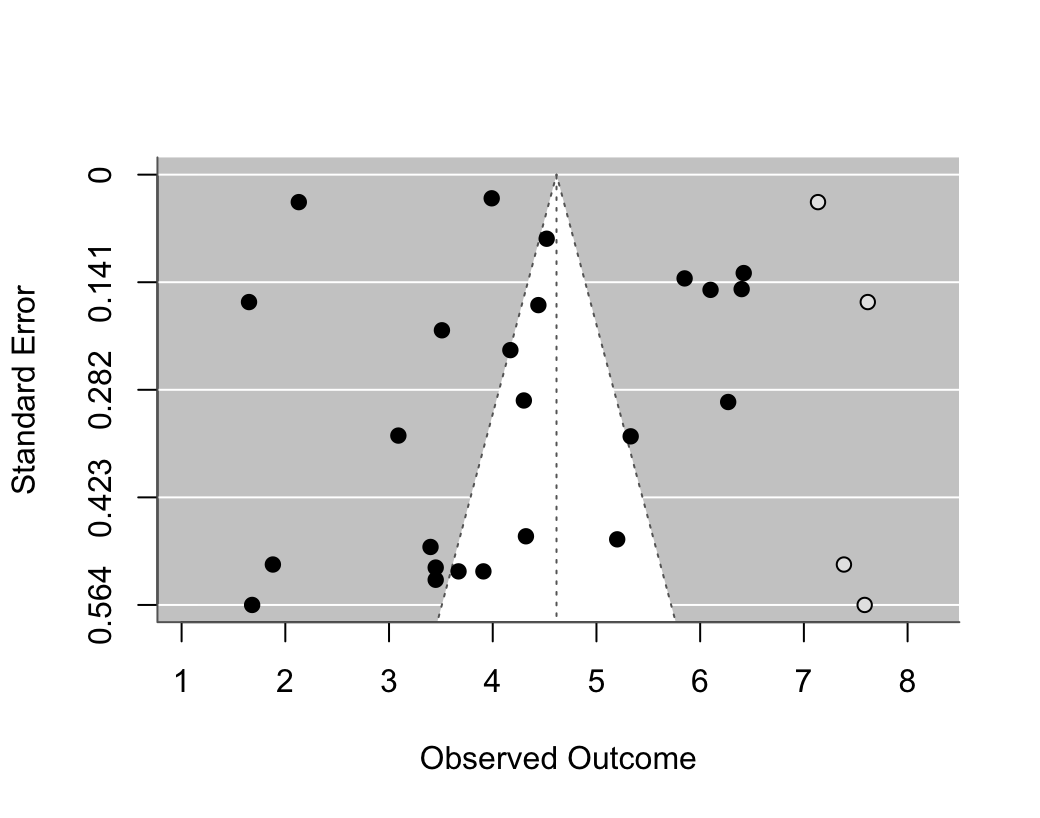


**Supplementary file 10**

sensitivity analyses for the transitivity of network

| excluded arm | RESD | τ2 | I2 (%) |
| --- | --- | --- | --- |
| BM | 0.46 (0.06, 1.17) | 2.59 | 100 |
| HW | 0.18 (0.01, 0.67) | 2.01 | 100 |
| NNS | 0.25 (0.03, 0.69) | 1.51 | 100 |
| ST | 0.33 (0.05, 0.88) | 1.46 | 100 |
| swaddling | 0.34 (0.05 0.91) | 1.44 | 100 |
| YT | 0.24 (0.02, .070) | 1.59 | 100 |

**Supplementary file 11**

the GRADE of these study

Table of reasons for downgrading

Based on all the above information, we GRADE each network estimate according to the following criteria.

(1) Study limitations: We downgraded by one level when the contributions from low RoB comparisons were less than 30% and contributions from moderate RoB comparisons were 70% or greater.

(2) Imprecision: We considered to downgrade the estimate if the MD point estimate is 0 or more and the lower limit of its CrI is below -0.5; or if the MD point estimate is less than 0 and the upper limit of its CrI is above 0.5.

(3) Inconsistency: we looked at the results of node split (6.2) and we downgraded the comparisons with important inconsistency (p<0.05).

(4) Indirectness: We have assured transitivity in our network by limiting the included studies to NIPS and newborn for pain management, and we further ran various sensitive analysis, assured that they did not violate transitivity of the network.

(5) Publication bias: The comparison-adjusted funnel plot and begger test suggested presence of overall publication bias. The review team decided by default to downgrade all the included studies for potential publication bias by one level.

|  | study limitation | imprecision | inconsistency | indirectness | publication bias | GRADE |
| --- | --- | --- | --- | --- | --- | --- |
| BM | Downgrade because >70% contribution from moderate RoB comparisons | No downgrade | No downgrade | No downgrade | downgrade | low |
| ST | No downgrade | No downgrade | No downgrade | No downgrade | downgrade | moderate |
| NNS | Downgrade because >70% contribution from moderate RoB comparisons | No downgrade | No downgrade | No downgrade | downgrade | low |
| YT | Downgrade because >70% contribution from moderate RoB comparisons | No downgrade | No downgrade | No downgrade | downgrade | low |
| swaddling | Downgrade because >70% contribution from moderate RoB comparisons | No downgrade | No downgrade | No downgrade | downgrade | low |
| HW | Downgrade because >70% contribution from moderate RoB comparisons | No downgrade | No downgrade | No downgrade | downgrade | low |
| ST VS BM | Downgrade because >70% contribution from moderate RoB comparisons | No downgrade | No downgrade | No downgrade | downgrade | low |
| NNS vs BM | Downgrade because >70% contribution from moderate RoB comparisons | No downgrade | No downgrade | No downgrade | downgrade | low |
| YT VS BM | Downgrade because >70% contribution from moderate RoB comparisons | No downgrade | No downgrade | No downgrade | downgrade | low |
| swaddling vs BM | Downgrade because >70% contribution from moderate RoB comparisons | No downgrade | No downgrade | No downgrade | downgrade | low |
| HW vs BM | Downgrade because >70% contribution from moderate RoB comparisons | No downgrade | No downgrade | No downgrade | downgrade | low |
| NNS vs ST | Downgrade because >70% contribution from moderate RoB comparisons | No downgrade | No downgrade | No downgrade | downgrade | low |
| YT vs ST | Downgrade because >70% contribution from moderate RoB comparisons | Downgrade because point estimate <0 but lower limit >0.5 | No downgrade | No downgrade | downgrade | very low |
| swaddling v s ST | Downgrade because >70% contribution from moderate RoB comparisons | No downgrade | No downgrade | No downgrade | downgrade | low |
| HW vs ST | Downgrade because >70% contribution from moderate RoB comparisons | No downgrade | No downgrade | No downgrade | downgrade | low |
| YT vs NNS | Downgrade because >70% contribution from moderate RoB comparisons | No downgrade | No downgrade | No downgrade | downgrade | low |
| swaddling vs NNS | Downgrade because >70% contribution from moderate RoB comparisons | Downgrade because point estimate <0 but higher limit >0.5 | No downgrade | No downgrade | downgrade | very low |
| HW vs NNS | Downgrade because >70% contribution from moderate RoB comparisons | No downgrade | No downgrade | No downgrade | downgrade | low |
| swaddling vs YT | Downgrade because >70% contribution from moderate RoB comparisons | No downgrade | No downgrade | No downgrade | downgrade | low |
| HW vs YT | Downgrade because >70% contribution from moderate RoB comparisons | No downgrade | No downgradel | No downgrade | downgrade | low |
| HW vs swaddling | Downgrade because >70% contribution from moderate RoB comparisons | No downgrade | No downgrade | No downgrade | downgrade | low |

BM=breastmilk, ST=sweet taste, HW=heel warming, YT=Yakson touch, NNS=non-nutritive sucking.

**Supplementary file 12**

the contribution figure


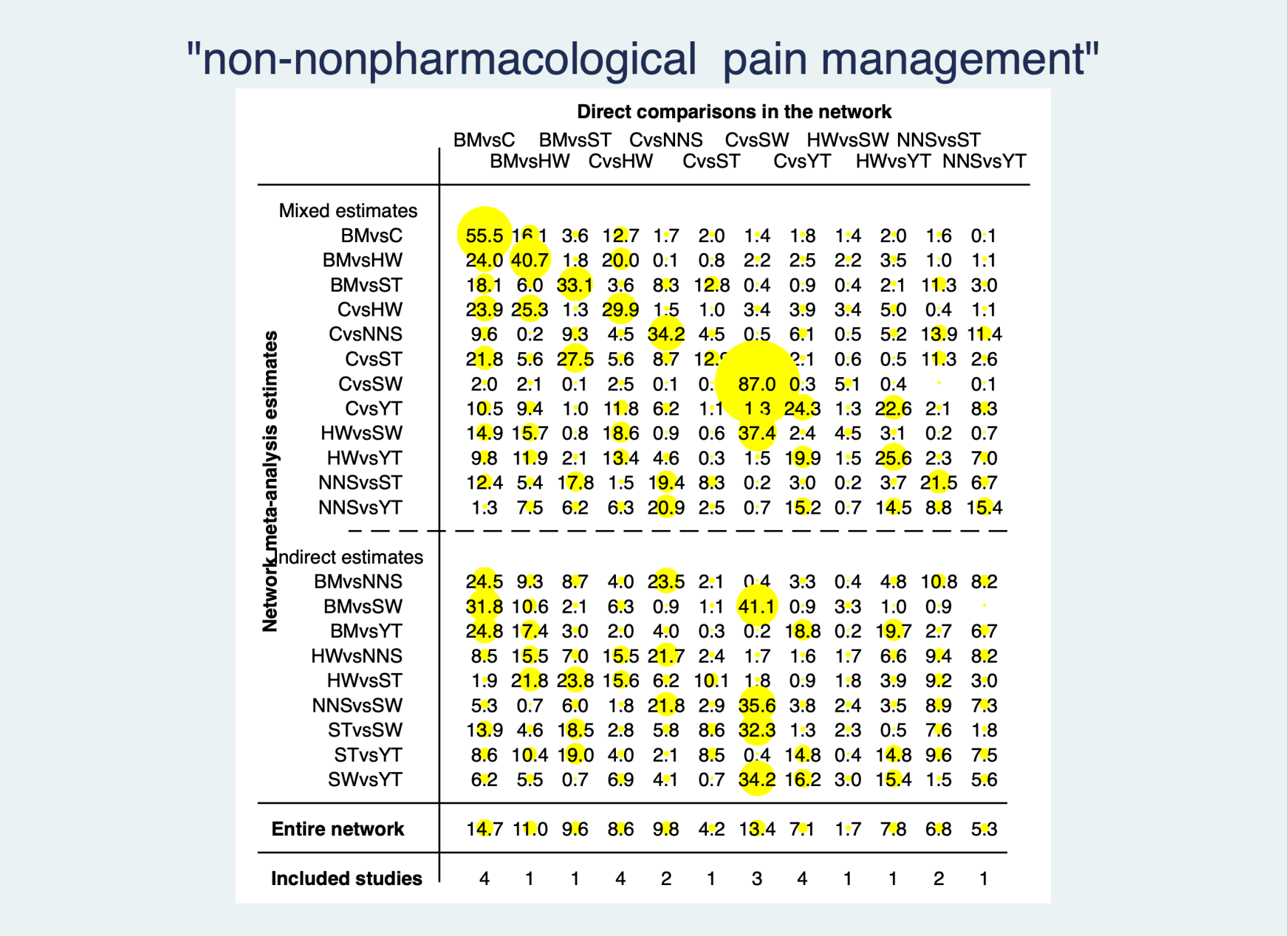

Supplement: Supplementary file 1 [file Table1.docx]
